# MDSCs Mediate Angiogenesis and Predispose Canine Mammary Tumor Cells for Metastasis via IL-28/IL-28RA (IFN-λ) Signaling

**DOI:** 10.1371/journal.pone.0103249

**Published:** 2014-07-30

**Authors:** Joanna Mucha, Kinga Majchrzak, Bartłomiej Taciak, Eva Hellmén, Magdalena Król

**Affiliations:** 1 Department of Physiological Sciences, Faculty of Veterinary Medicine, Warsaw University of Life Sciences – WULS, Warsaw, Poland; 2 Department of Animal Environment Biology, Faculty of Animal Sciences, Warsaw University of Life Sciences – WULS, Warsaw, Poland; 3 Department of Anatomy, Physiology and Biochemistry, Swedish University of Agricultural Sciences, Uppsala, Sweden; Yong Loo Lin School of Medicine, National University of Singapore, Singapore

## Abstract

**Background:**

Myeloid-derived suppressor cells (MDSCs) function in immunosuppression and tumor development by induction of angiogenesis in a STAT3-dependent manner. Knowledge of MDSC biology is mainly limited to mice studies, and more clinical investigations using spontaneous tumor models are required. Here we performed *in vitro* experiments and clinical data analysis obtained from canine patients.

**Methods:**

Using microarrays we examined changes in gene expression in canine mammary cancer cells due to their co-culture with MDSCs. Further, using Real-time rt-PCR, Western blot, IHC, siRNA, angiogenesis assay and migration/invasion tests we examined a role of the most important signaling pathway.

**Results:**

In dogs with mammary cancer, the number of circulating MDSCs increases with tumor clinical stage. Microarray analysis revealed that MDSCs had significantly altered molecular pathways in tumor cells *in vitro*. Particularly important was the detected increased activation of IL-28/IL-28RA (IFN-λ) signaling. The highest expression of IL-28 was observed in stage III/IV mammary tumor-bearing dogs. IL-28 secreted by MDSCs stimulates STAT3 in tumor cells, which results in increased expression of angiogenic factors and subsequent induction of angiogenesis by endothelial cells, epithelial-mesenchymal transition (EMT) and increased migration of tumor cells *in vitro*. Knockdown of IL-28RA decreased angiogenesis, tumor cell invasion and migration.

**Conclusions:**

We showed for the first time that MDSCs secrete IL-28 (IFN-λ), which promotes angiogenesis, EMT, invasion and migration of tumor cells. Thus, IL-28 may constitute an interesting target for further therapies. Moreover, the similarity in circulating MDSC levels at various tumor clinical stages between canine and human patients indicates canines as a good model for clinical trials of drugs targeting MDSCs.

## Introduction

Myeloid-derived suppressor cells (MDSCs) are associated with tumor progression. Transplantation of MDSCs into tumor-bearing mice significantly promoted tumor growth [1], [2], whereas administration of MDSCs after 5-fluorouracil treatment completely abolished the anti-tumor effect of the drug [3]. Injection of antibodies targeting MDSCs in tumor-bearing mice inhibited tumor growth and reduced cancer metastasis [4]-[7]. The proposed mechanism of MDSCs action is induction of immunosuppression and promotion of angiogenesis by production of vascular endothelial growth factor (VEGF) [8]. MDSC promotion of angiogenesis is driven by activated signal transducer and activator of transcription 3 (STAT3) (9). However, the signaling by which MDSCs activate STAT3 in cancer cells has not been fully elucidated.

Anti-MDSC treatment has been successfully used as a part of anti-cancer therapy. Treatment of tumor-bearing mice with drugs that target MDSCs, such as gemcitabine, delays tumor progression, improves survival, and enhances efficacy of cancer vaccines and immunotherapies [10]–[12]. Moreover, reduction of a number of MDSCs in mice facilitated the reduction of established metastatic disease after the removal of primary tumors [7], [13].

Dogs are excellent models for human cancer studies, as they naturally develop the same tumors as humans with an intact immune system and with a syngeneic host and tumor microenvironment [14], [15]. Moreover, both species share similar environmental, nutrition, age, sex, and reproductive factors that lead to the development and progression of cancers [16]. Dog tumors are histologically similar to human tumors, and many histological types of cancer are associated with similar genetic alterations in both species. Dogs also respond similar to humans to conventional therapies. Based on the similar activity of P450 cytochrome in both species and shorter duration time of canine clinical trials, the canine model can be used in clinical trials of human drugs. For example, the disease-free time interval in dogs treated for cancer is 18 months, compared with 7 years needed to assess treatment outcomes in humans [17]. Thus, results obtained in dogs can provide an opportunity to answer some questions that are unanswered using standard preclinical and clinical models. In 2005/2006, the “Comparative Oncology” program was launched in the USA with the aim of introducing the canine model into mainstream cancer research, mainly as an intermediate model between rodents and humans in clinical trials of anticancer drugs [17].

The aim of our study was two-fold: to assess the number of MDSCs in healthy and tumor-bearing dogs and to compare these results to human studies. These findings may be very useful for further introduction of the canine model in clinical trials of drugs targeting MDSCs. The second aim was to elucidate the mechanism by which MDSCs increase STAT3 activation in cancer cells. As the overall cellular role of MDSCs in cancer cells has not been fully established, targeting of molecule that activates STAT3 instead of using chemotherapeutic agents against MDSCs could constitute a better approach to cancer treatment. Thus far, anti-MDSCs therapy has been used experimentally only in mice. Before introduction of this type of therapy into human patients, it would be useful to employ the canine model. Following the aims of “Comparative Oncology” trials, this approach could reduce the risk of side effects related to drugs and ensure their early elimination from later phases of human clinical development. Furthermore, it could result in fewer human participants entering trials with potentially ineffective or unsafe drugs [16].

## Materials and Methods

### Canine mammary tumor cell lines and culture

Cell lines were described in our previous studies [18], [19]. In brief, the anaplastic cancer cell line P114 was kindly donated by Dr. Gerard Rutteman (Utrecht University, The Netherlands). A simple carcinoma cell line (CMT-U27) and a spindle cell mammary tumor cell line (CMT-U309) were established by Prof. Dr. Eva Hellmén (Swedish University of Agricultural Sciences, Sweden). Cells were cultured in RPMI 1640 medium supplemented with 10% (v/v) heat-inactivated fetal bovine serum, penicillin-streptomycin (50 IU/mL), and fungizone (2.5 mg/mL; Sigma-Aldrich, USA) in an atmosphere of 5% CO_2_ and 95% humidified air at 37°C [19], [20]. The human umbilical venous endothelial cell line (HUVEC) was purchased from Life Technologies (USA). HUVECs were cultured in 200PRF medium (Gibco, USA) with Low Serum Growth Supplement (LSGS) (Gibco) in standard culture conditions. For the 3D tubule formation assays, early passages were used (P1–P4).

### MDSCs and T lymphocyte analysis

Anti-coagulated whole blood was collected for diagnostic purposes from healthy dogs (n = 26) or mammary tumor-bearing dogs (n = 27) during routine veterinarian procedures. Then, it was immediately subjected to mononuclear cell separation using Accuspin System-Histopaque 1077 (Sigma-Aldrich), according to the manufacturer's protocol. The dogs' owners gave written or verbal permission for the use of their animals' blood for this work. Samples were obtained within study approved by the III Local Ethical Committee (approval no. 8/2012, 17.01.2012) of the Warsaw University of Life Sciences. Mammary tumor-bearing dogs were classified based on the clinical data of tumor size (T designation), presence of lymph node metastases (N category) and of distant metastases (M category), according to the original World Health Organization's (WHO) TNM system [20]. For this study, blood from mammary tumor-bearing dogs in the following stages was used: stage I (n = 11), stage II (n = 4), stage III (n = 4) and stage IV (n = 8). Isolated mononuclear blood cells were incubated for 1 h at room temperature with canine-specific rat monoclonal anti-Gr1 (RPE conjugated), anti-CD11b (FITC conjugated) and mouse monoclonal anti-CD33 (APC conjugated) antibodies (all obtained from Thermo Scientific, USA), according to the manufacturer's instructions. Leukocytes were initially identified and gated on the basis of morphological criteria (SSC vs. FSC cytogram) using FACSAria II (BD Biosciences, USA). Subsequently, Gr1/CD11b/CD33-positive cells were gated, counted and sorted for the purposes of RNA isolation or co-culture with cancer cells in trans-well chambers.

### Co-culture

Canine mammary tumor cells were seeded in 6-well plates, and MDSCs sorted from the blood of healthy dogs were added to the transwell inserts at a 1∶5 ratio to tumor cells. The co-culture was maintained for 48 h.

### siRNA transfection and IL28 treatment

The siRNA transfection procedure used in canine mammary tumor cells was described in detail in our previous study [19]. The canine (*Canis lupus familiaris*) *Il-28ra* sequence was obtained from Gene Bank (accession number: XM_850017.3). The siRNA duplexes were designed by Sigma-Aldrich and two duplexes were chosen for further experiments. The duplex sequences are as follows: the first duplex, CUCGAAUUCUCCAACGACAdTdT and UGUCGUUGGAGAAUUCGAGdTdT; and the second duplex, AUCACCAGGGCUGAAUAUAdTdT and UAUAUUCAGCCCUGGUGAUdTdT. For *Il-28ra* silencing, a mixture of both duplexes was used (30 pmol+30 pmol) with Lipofectamine 2000 (Life Technologies) at concentrations recommended by the manufacturer. All experiments with transfected cells were conducted 48 h after the transfection. Mock transfected cells were used as controls (transfected with Lipofectamine 2000 and a non-coding siRNA sequence obtained from Life Technologies).

For IL-28 (Bio-Rad, USA) treatment, cells were seeded in normal culture medium supplemented with 100 U/ml [21] of the protein for 48 h. The medium was replaced with fresh medium containing IL-28 every 24 h.

### Microarray analysis

Total RNA (t-RNA) was isolated from samples using an RNA kit (A&A Biotechnology, Poland), according to the manufacturer's protocol. The quantity of t-RNA was measured using a NanoDrop instrument (NanoDrop Technologies, USA), and the final RNA quality and integrity were assessed using a BioAnalyzer (Agilent, USA). Only high-quality samples (RIN >8) were used in further analyses.

The Quick Amp Labeling Kit (Agilent) was used to amplify and label target RNA to generate complementary RNA (cRNA) for oligo microarrays used in gene expression profiling and other downstream analyses. The gene expression of neoplastic cell lines, grown under co-culture conditions with MDSCs, was compared against the gene expression of the same neoplastic cell line grown in monoculture. Each sample was examined in a dye-swap to eliminate the effect of label factor. The hybridization was performed with canine-specific AMADID Release GE 4x44K microarrays (Agilent) using the Gene Expression Hybridization Kit (Agilent) according to the manufacturer's protocol. Acquisition and analysis of hybridization intensities were performed using a DNA microarray scanner (Agilent), and data were extracted using Agilent's Feature Extraction software with normalization and robust statistical analyses.

### Biostatistical analysis

Statistical analyses were performed using Gene Spring software (Agilent) and BRB ArrayTools (http://linus.nci.nih.gov/BRB-ArrayTools.html, Biometric Research Branch, US National Cancer Institute).

Intensities were normalized using average factors scaled to the median array intensities over the entire array using the median array as a reference. Probe sets that yielded a maximal normalized nonlog intensity value of 10 or less were filtered out from further analysis. The mRNAs that were differentially expressed between signal and control samples (*P*<0.05; FC>2.0) were identified by class comparison analysis using two-sided Student t-tests followed by bootstrapping. The mRNAs that were significantly regulated by co-culture conditions in all neoplastic cell lines were selected from six technical and three biological repetitions. Areas of these analyses have been deposited in NCBI's Gene Expression Omnibus and are accessible using the GEO Series accession number GSE53373. Genes and corresponding signaling pathways were identified using PANTHER pathway analysis software [22].

### Real-time RT-PCR

Sequences of key genes were obtained from the NCBI database. Primers were designed using Primer3 software (free online access) and were checked using Oligo Calculator (free online access) and Primer-Blast (NCBI database). Primer sequences are listed in Table 1. The housekeeping gene *Rps 19* was used as internal control [23], [24]. Quantitative RT-PCR was performed using a fluorogenic Lightcycler Fast Strand DNA SYBR Green kit (Roche) and a Light Cycler (Roche). Data were analyzed using the comparative Ct method [26]. The experiment was repeated five times. PCR products were electrophoresed through ethidium bromide-stained 2% agarose gels (Sigma-Aldrich) for 60 min at 90 mV in Tris-borate-EDTA buffer. The gels were then visualized under UV light.

**Table 1:** Primer's sequences used in this study and their annealing optimal temperature and time.

Gene name	Left Primer	Right Primer	Annealing temp	Annealing time
*Il18*	GTGATGAAGGCCTGGAATCAGA	CTGTACAGTCAGAATCGGGCA	62	6
*Il-28ra*	TAGAAGGTGGCGAAAAGTGA	CTGGCTCCACTTCAAAAAGGTA	59	8
*Il-28*	GCTGACCGTGACTGGAGC	GACAGGGACTTGAACTGGGCTA	63	4
*Sema6C*	CGGATTTCCAGGCCAGTGAT	AGTCGTACTTGGCAGAACGG	60	4
*Krt17*	GCCTGTCTGGGAAGTGGAAG	TGCGTGTCTCTGGTCTCAAG	63	7
*Mmp20*	ACAAGTACCACCTCGCACAG	AAGGCTTGCAGCTCCTTGAT	62	6
*Rps19*	CCTTCCTCAAAAAGTCTGGG	GTTCTCATCGTAGGGAGCAAG	61	10

The mRNA sequences of key genes were obtained from NCBI database. Primers were designed using PRIMER3 software (free on-line access) and checked using Oligo Calculator (free on-line access) and Primer-Blast (NCBI database).

### Western blotting

Protein extracts from cultured cells (control cells, cells treated with silenced IL-28RA expression and cells treated with IL-28) were lysed with RIPA buffer (Sigma-Aldrich) supplemented with protease inhibitor cocktail (Sigma-Aldrich) and phosphatase inhibitor cocktail (Sigma-Aldrich). Total protein concentrations in lysates were determined using a Bio-Rad protein assay (Bio-Rad Laboratories Inc.). Proteins (50 µg) were resolved using SDS-PAGE and transferred onto PVDF membranes (Sigma-Aldrich). The membranes were then blocked with 5% non-fat dry milk in TBS buffer containing 0.5% Tween 20. The membranes were incubated overnight with the primary anti-canine antibodies (or antibodies showing cross-reactivity with canine) anti-IL28RA (rabbit, Aviva Systems Biology), anti-p-STAT3 (rabbit, Thermo Scientific), anti-STAT3 (rabbit, Thermo Scientific), anti-VEGF-C (rabbit, Thermo Scientific), anti-IL-18 (goat, Santa Cruz), anti-SEMA3B (rabbit, Santa Cruz) and anti-β-actin (mouse, Santa Cruz) at 4°C. The membranes were washed three times in TBS containing 0.5% Tween 20 and incubated for 1 h at room temperature with secondary antibodies conjugated with the appropriate infrared (IR) fluorophore IRDye 800 CW or IRDye 680 RD at a dilution of 1∶5000. An Odyssey Infrared Imaging System (LI-COR Biosciences, USA) was then used to analyze protein expression. Scan resolution and intensity of the instrument were set at 169 µm and 4, respectively. Quantification of the integrated optical density (IOD) was performed using the analysis software provided with the Odyssey scanner (LI-COR Biosciences). To remove antibodies, the membranes were incubated for 15 min at room temperature in Restore Western Blot Stripping Buffer (Thermo Scientific, USA). This experiment was repeated five times.

### Culture on Matrigel matrix

Culture plates (35 mm; Corning Inc.) were coated with 100 µL of growth factor-reduced Matrigel (BD Biosciences) and were left to solidify for 30 min at 37°C. The control cells, siRNA-treated cells or IL-28 treated cells were then plated at a concentration of 10^4^ cells/mL and cultured for 24 hrs. Cell growth on Matrigels was observed using a phase contrast microscope.

### Invasion and migration assay

The BD BioCoat 24-Multiwell Invasion System (BD Biosciences) pre-coated with BD Matrigel Matrix was used according to the manufacturer's protocol. The insert plates were prepared by rehydrating the BD Matrigel Matrix layer with phosphate buffered saline (PBS) for two hours at 37°C. The rehydration solution was then carefully removed and 500 µl of cell suspension (control cells, cells with IL-28RA knockdown or cells treated with IL-28) in RPMI 1640 medium containing 0.2% FBS was added to the apical chambers (2.5×10^5^ cells). Then, 750 µl of chemoattractant (20% FBS) was added to each of the basal chambers. As a negative control for background reduction, culture medium without cells was used. Assay plates were incubated for 22 h at standard culturing conditions. Incubation medium was carefully removed from the apical chamber and insert system was transferred into a second 24-well plate containing 500 µl of 2.5 µg/ml Calcein AM in Hanks' Balanced Salt solution (HBSS). Plates were incubated for 1 h at standard culturing conditions. The fluorescence of invaded cells was measured at excitation wavelength 485 nm and emission wavelength 530 nm using a florescent plate reader with bottom reading capabilities, Infinite 200 PRO Tecan (TECAN, Switzerland). To visualize the invaded cells, a fluorescence microscope (Olympus BX60) at 4× magnification was used. The experiment was repeated three times.

To evaluate migratory potential, the BD Falcon FluoroBlock 24-Multiwell Insert Plates (8 micron pore size) (BD Biosciences) were used. The determination protocol for the canine mammary cancer cell migration was the same as the invasion assay, with the exception that no Matrigel was used and rehydrating of the plate was omitted. All samples were assayed three times.

### Tubule formation assay (angiogenic *in vitro* assay)

The 3D tubule formation by human endothelial cells (HUVECs) initiated by cancer cells originating from other species has been previously established [26]. We applied this model for our experiments using canine mammary tumor cells. Culture plates (24 wells; Corning Inc.) were coated with Growth-Factor Reduced Matrigel Matrix (BD Biosciences) and allowed to solidify for 30 min at 37°C. The HUVECs were then plated at a concentration of 4.2×10^4^ cells/cm^2^ according to the manufacturer's instructions. We used a positive inducer control, LSGS-supplemented Medium 200PRF, and a negative control, LSGS-supplemented medium with 30 µM Suramin (Sigma-Aldrich). We also assessed the influence on tubule formation of standard tumor cell culture medium and culture medium supplemented with 100 U/mL of IL-28. Canine mammary tumor cells were plated in trans-well inserts as follows: control cells, cells silenced for IL-28RA, cells treated with IL-28 (100 U/mL) for 48 h before the experiment, and cells treated with IL-28 (100 U/mL) for 48 h before the experiment and during the experiment. HUVEC reorganization into 3D vessel structures was examined after 6 h using a phase contrast microscope. The experiment was repeated three times, and each experimental condition was assayed in duplicate (n = 6).

### Immunohistochemical (IHC) examination

Cell lines (control cells, cells with IL-28RA knockdown, and cells treated with IL-28) cultured on Lab-Tek 8-chamber culture slides (Nunc Inc., USA) were subjected to IHC analysis after ethanol (70%) fixation for 10 min. Samples were incubated in a peroxidase blocking reagent (Dako, Denmark) for 10 min at room temperature prior to 30 min incubation in 5% bovine serum albumin (Sigma-Aldrich). Cell lines were incubated with 1∶100 dilution of anti-cytokeratin (clone AE1/AE3) or anti-vimentin antibodies for 1 h at room temperature. The Envision kit comprising labeled polymers of secondary anti-rabbit/mouse antibodies conjugated with the HRP enzyme complex (Dako) was used. The 3,3′-diaminobenzidine (Dako) substrate was used to develop colored products. Finally, nuclei were counterstained with hematoxylin. Each IHC experiment was controlled by omitting primary antibodies. Four slides of each cell line were analyzed. Pictures of each slide were taken using an Olympus BX60 microscope. Colorimetric intensities of IHC-stained antigen spots (brown precipitate reflecting antigen expression) were counted using a computer-assisted image analyzer (Olympus Microimage Image Analysis version 4.0 software for Windows, USA). The intensities of color related to each antigen spot were expressed as mean pixel IOD.

### Statistical analysis

Statistical analyses were performed using Prism version 5.00 software (GraphPad Software, USA). Two-way analysis of variance (ANOVA), ANOVA with Tukey's honest significant difference post-hoc test were applied, and differences were considered significant when *P*<0.05 or highly significant when *P*<0.01 or *P*<0.001.

## Results

### Number of circulating MDSCs in the blood of canine patients and the effect of co-culture with canine mammary tumor cells *in vitro*

**Figure 1:**
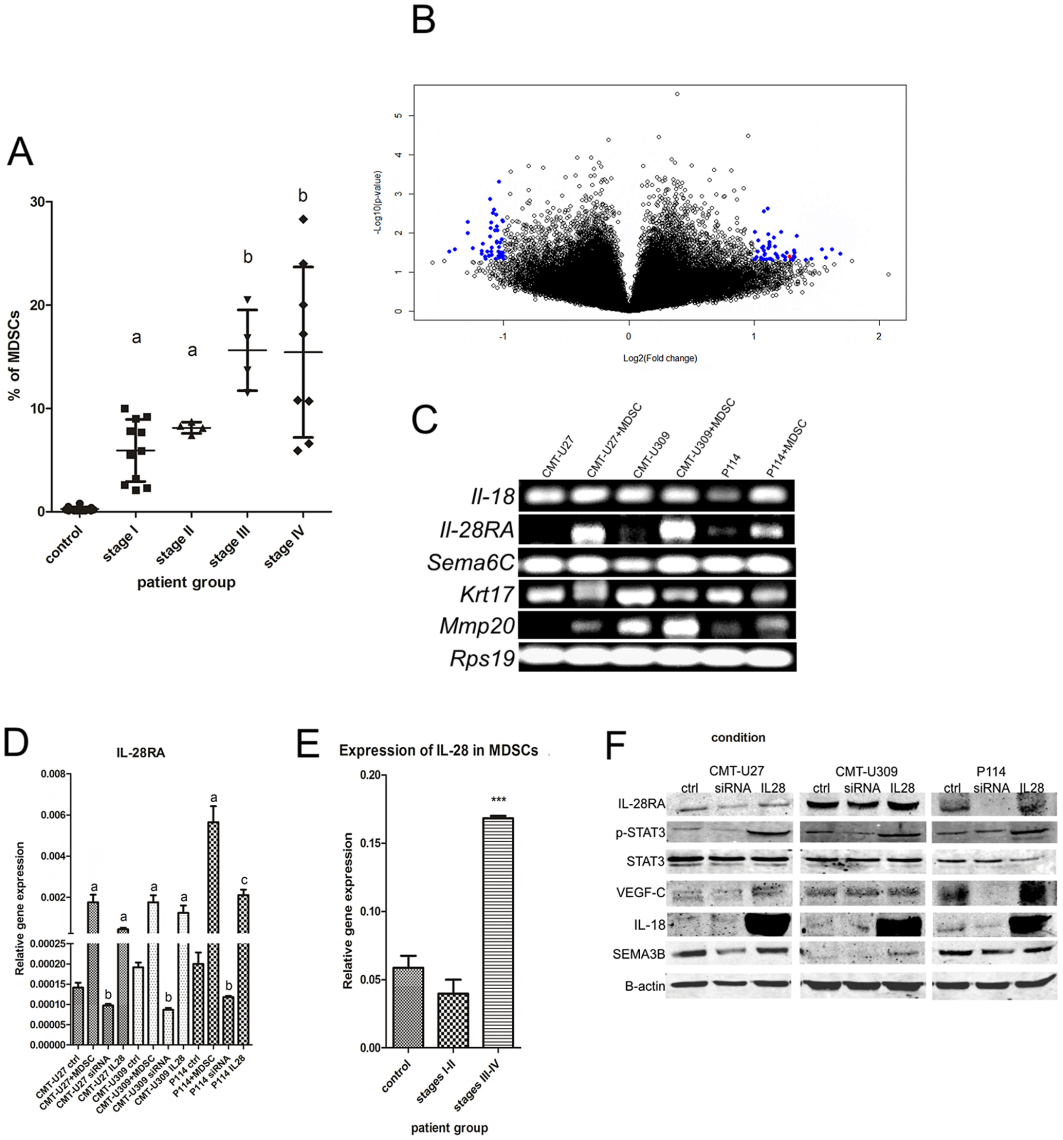
Number of MDSCs in mammary tumor-bearing dogs, their IL-28 expression and changes in canine mammary tumor cells gene/protein expression due to their co-culture with MDSCs. (A). The number of circulating MDSCs (%) within white blood cells in healthy donors, and dogs with stage I, II, III and IV mammary cancer. Results that differed significantly compared to control are marked by ‘a’ (*P*<0.01) or ‘b’ (*P*<0.001). (B). Volcano plot of gene expression in canine mammary tumor cells co-cultured with MDSCs compared to monocultured cells. Genes which expression differed significantly (*P*<0.05, FC>2.0) are marked by blue dots. *Il-28ra* is marked as a red dot. The plot was generated using BRB software. (C). Changes in expression of selected genes in canine mammary tumor cells due to co-culture with MDSCs visualized on agarose gel using UV light (ctrl, control cells grown as mono-culture; +MDSC, cells grown as co-culture with MDSCs). (D). Relative *Il-28ra* gene expression in canine mammary tumor control cells transfected with non-coding siRNA (ctrl), cells co-cultured with MDSCs (+MDSC), transfected with *Il-28ra*-specific siRNA (siRNA) and treated with IL-28 (IL28). Results that differ significantly compared to control are marked as ‘a’ (*P*<0.001), ‘b’ (*P*<0.05) or ‘c’ (*P*<0.01). (E). Relative gene expression of *Il-28* in MDSCs isolated from blood of healthy dogs, and dogs with stage I/II or III/IV mammary cancer. (F). Expression of selected target and downstream signaling proteins in control cells mock-transfected (ctrl), cells with knockdown of IL-28RA expression (siRNA) and cells treated with IL-28 (IL28).

FACS analysis showed that the number of MDSCs (CD11b+/Gr1+/CD33+) in the leukocyte population in the blood of healthy dogs was 0.35 (SD = 0.48). In dogs with stage I or II mammary cancer, MDSC number was significantly higher (*P*<0.01): 5.94 (SD = 2.99) and 8.13 (SD = 0.54), respectively. However, the number of MDSCs in dogs with stage III and IV of mammary cancer was the highest (*P*<0.001): 15.63 (SD = 3.91) and 15.44 (SD = 2.92), respectively (Fig. 1A).

Analysis of gene expression in canine mammary tumor cell lines co-cultured with MDSCs showed 107 significantly upregulated genes and 30 significantly downregulated genes due to co-culture (Table S1, Fig. 1B). All significantly regulated genes and their fold change are listed in Table S1. Expression analysis of selected genes (*Il-18, Il-28ra, Sema6C, Krt17, Mmp20*) using real-time RT-PCR confirmed the same trends as the microarray experiment (Fig. 1C, Fig. S1A, Table S1).

PANTHER analysis showed that among the upregulated genes, most were involved in inflammation mediated by cytokine and chemokine signaling pathways, cytoskeleton regulation by Rho GTPase, interleukin signaling pathway and Wnt signaling pathway.

After careful analysis of the gene list (Fig. 1B, Table S1) and their functions, we focused on analyzing the role of IL-28/IL-28RA (IFN-λ) in a “dialogue” between MDSCs and tumor cells and their role in tumor cell biology. According to the literature, IL-28 regulates STAT3 signaling via IL-28RA [27]. STAT3 is activated in cancer cells by MDSCs [9]. Thus, our working hypothesis was that IL-28 functions an intermediate protein in the signaling between MDSCs and tumor cells, which activates STAT3 in neoplastic cells and leads to cancer metastasis and angiogenesis.

Using real-time RT-PCR, we observed that co-culture of MDSCs with CMT-U27 canine mammary cancer cells increased *Il-28ra* expression 12.44-fold (*P*<0.001). In the CMT-U309 cell line, co-culture with MDSCs increased *Il-28ra* expression 9.17-fold (*P*<0.001), whereas in the P114 cell line, expression increased 28.29-fold (*P*<0.001) (Fig. 1D). Similarly, treatment of cell lines with IL-28 significantly (*P*<0.001) increased *Il-28ra* expression 3.2-fold, 6.52-fold and 10.57-fold in CMT-U27, CMT-U309 and P114 cell lines, respectively (Fig. 1D). Treatment of CMT-U27 cells with siRNA targeting *Il-28ra* significantly decreased IL-28ra expression to 30% of expression in control cells. In CMT-U309 and P114 cells lines, we observed a 45% and 41% decrease in *Il-28ra* expression, respectively, due to siRNA treatment (Fig. 1D). Because no differences were observed between control cells and mock-transfected cells, all control cells in further experiments were mock transfected.

We also found that *Il-28* expression in MDSCs of mammary tumor-bearing dogs with stage III/IV mammary cancer was 2.87-fold higher *(P<*0.001) than in MDSCs of healthy dogs, and 4.24-fold higher (*P*<0.001) than in MDSCs of patients with stages I–II of mammary cancer (Fig. 1E).

These results as well as the effect on downstream signaling were confirmed using Western blot analysis (Fig. 1F, Fig. S1B). We confirmed that treatment of all cell lines with siRNA-targeting *Il-28ra* decreased IL-28RA expression whereas treatment with IL-28 increased IL-28RA expression. IL-28RA expression level reflected STAT3 phosphorylation status, with a lower level of STAT3 phosphorylation in *Il-28ra*-siRNA treated cells and a higher level of phosphorylation in IL-28 treated cells. We also observed that in cells with silenced IL-28RA, the levels of VEGF-C, IL-18 and SEMA3B decreased. However, in cells treated with IL-28, the levels of all three proteins increased (Fig. 1F, Fig. S1B).

### IL-28/IL-28RA signaling enhances angiogenesis in vitro

**Figure 2:**
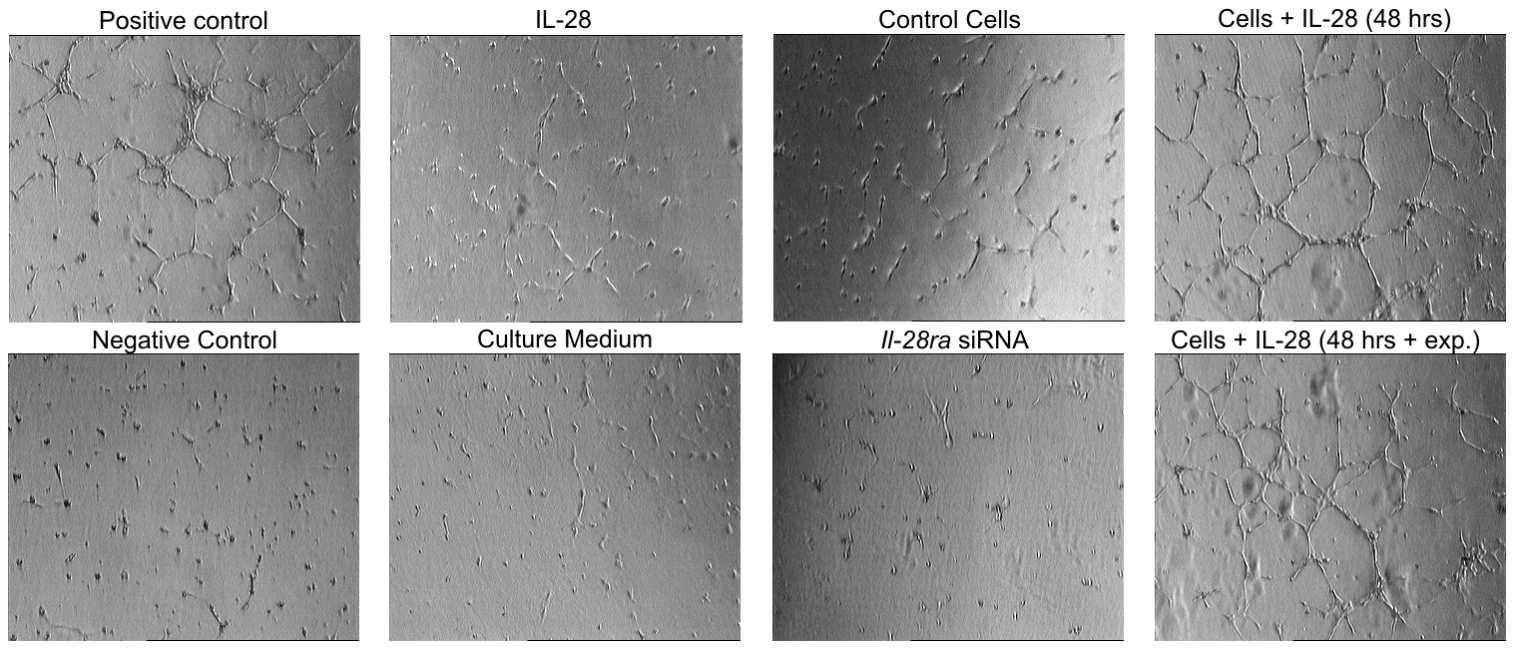
Vessel formation (3D) by HUVECs. Representative pictures showing 3D vessel formation by HUVECs due to co-culture with P114 canine mammary control cells (mock-transfected), cells treated with *Il-28ra*-specific siRNA, cells treated with IL-28 (before the experiment), cells treated with IL-28 during the experiment and positive and negative control cells, as well as only IL-28 treatment.

The angiogenic *in vitro* assay showed that HUVECs stimulated by IL-28 (cell treated with IL-28 for 48 h before experiment and cells treated with IL-28 before and during the assay) for 6 h formed 3D vessels (Fig. 2) as similarly observed in positive controls. Stimulation of HUVECs with control tumor cells or with IL-28 administered alone (without tumor cells) for 6 h induced only small branching of endothelial cells (Fig. 2). However, stimulation of HUVECs by cells transfected with *Il-28ra-*specific siRNA for 6 h did not induce angiogenesis (similar to the negative control).

### IL-28/IL-28RA signaling promotes epithelial-mesenchymal transition (EMT)

**Figure 3:**
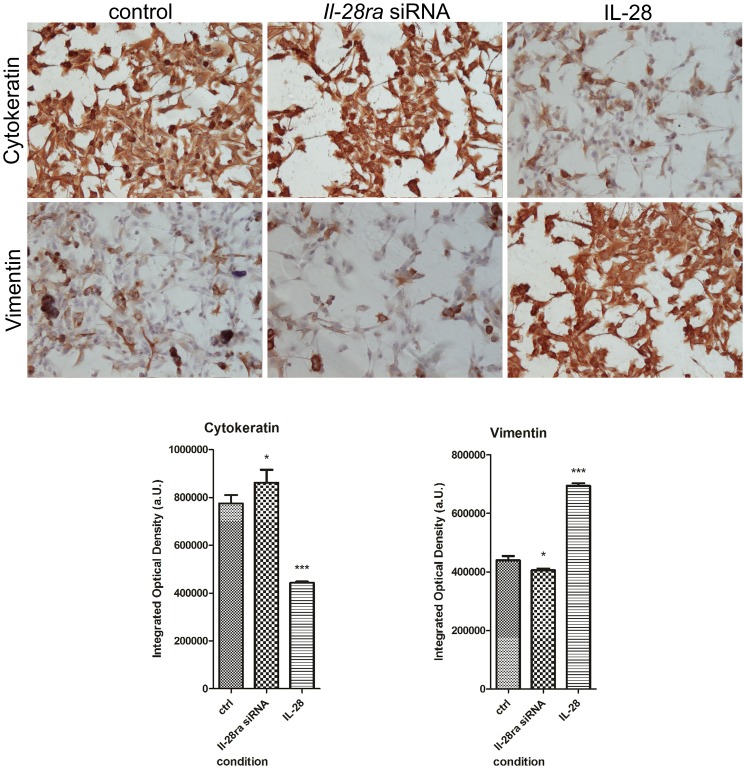
Changes in cytokeratin and vimentin expression. Representative pictures showing changes in expression of cytokeratin (upper panel) and vimentin (bottom panel) in P114 canine mammary control cells (mock-transfected), cells treated with *Il-28ra*-specific siRNA and cells treated with IL-28. Values that differed significantly are marked as * (*P*<0.05) or *** (*P*<0.001).

Immunohistochemical analysis showed that canine mammary tumor cells undergo EMT due to treatment with IL-28. In control conditions, cytokeratin expression was very strong in all the examined cell lines (mean IOD = 750,000 a.U.), whereas vimentin expression was weak (IOD = 433,000 a.U.) (Fig. 3). We observed that because of IL-28 treatment, cytokeratin expression significantly decreased (mean IOD = 424,000 a.U., *P*<0.001) whereas vimentin expression significantly increased (mean IOD = 723,000 a.U., *P*<0.001) (Fig. 3). These differences were not as highly significant due to IL-28RA knockdown (*P*<0.05). However, the mean IOD of cytokeratin was 862,000 a.U., and vimentin 405,781 a.U. (Fig. 3).

**Figure 4:**
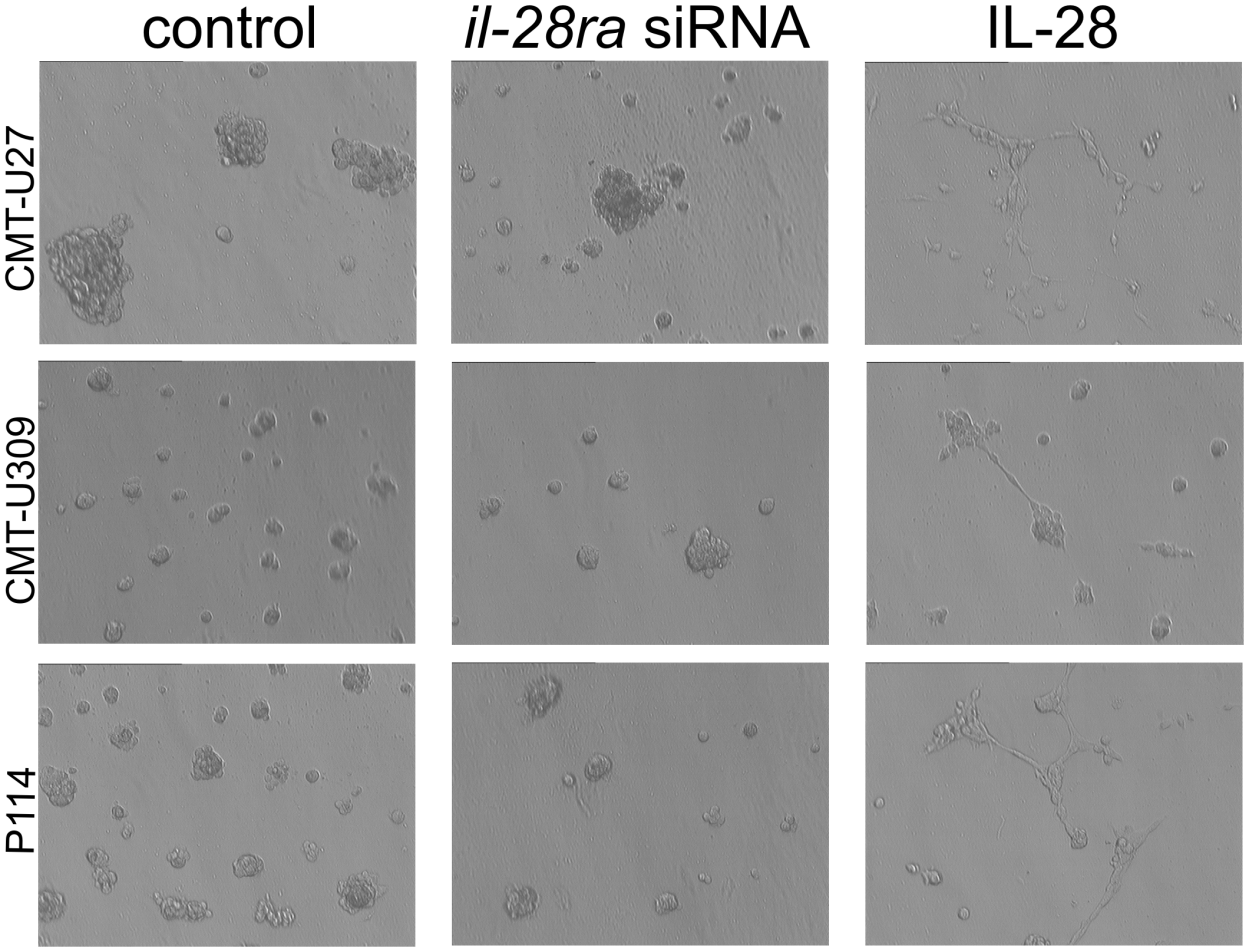
Growth characteristics on Matrigel matrix. Phase contrast micrographs of CMT-U27, CMT-U309, and P114 cells grown on the Matrigel matrix under control conditions or treated with *il-28ra* siRNA either IL-28. Control and siRNA-treated neoplastic cells formed colonies, whereas those treated with IL-28 changed their shape for spindle-like, invaded the Matrigel matrix and formed branches.

Analysis of changes in tumor growth patterns on Matrigel matrix showed that control cells or siRNA-treated cells formed unbranched colonies. In contrast, incubation with IL-28 (Figure 4) led to the formation of branches and invasion of the Matrigel matrix.

### IL-28/IL-28RA signaling enhances canine mammary tumor invasion and migration

**Figure 5:**
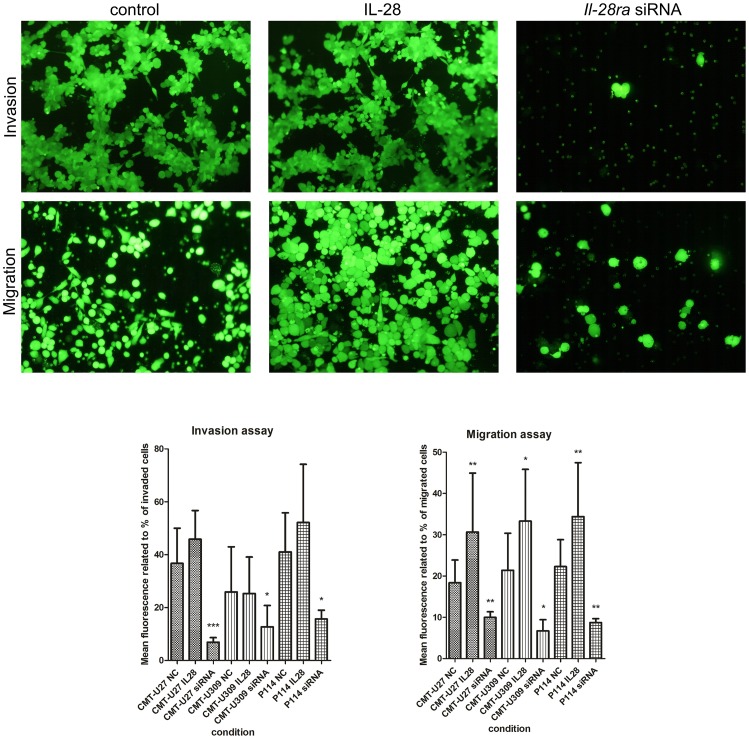
Migration and invasion *in vitro* assay. Representative pictures showing invaded (upper panel) and migrated (bottom panel) CMT-U27 canine mammary tumor cells: control (mock-transfected) (NC), treated with IL-28 (IL28) or treated with *Il-28ra*-specific siRNA (siRNA). Values that differed significantly are marked as * (*P*<0.05), ** (*P*<0.01) or *** (*P*<0.001).

Invasion and migration assays in Boyden chambers showed a significant role of IL-28 in both processes. Silencing of IL-28RA significantly decreased the number of invading cells in all of the examined cell lines (Fig. 5). In CMT-U27 cell line, this effect was 5.33-fold (*P*<0.001), in the CMT-U309 cell line 2-fold (*P*<0.05), and in the P114 cell line, it was 2.62-fold (*P*<0.05) (Fig. 4) based on the fluorescence related with the number of invading cells. However, microscopic examination showed almost complete inhibition of cell invasion after IL-28RA knockdown (Fig. 5). Supplementation of medium with IL-28 did not cause any significant effect on cell invasion. However, IL-28 treatment as well as IL-28RA silencing significantly affected cell invasion. In the CMT-U27 cell line, treatment with IL-28 increased migration 1.67-fold (*P*<0.01) and IL-28RA knockdown caused a 1.84-fold decrease of their migratory abilities (*P*<0.01) (Fig. 5). In the CMT-U309 cell line, IL-28 increased migratory abilities 1.56-fold (*P*<0.05), however IL-28RA silencing decreased migration 3.19-fold (Fig. 5). In the P114 cell line, we observed 1.54-fold increased migration due to IL-28 treatment and 2.56-fold decreased migration due to transfection with *Il-28ra*-specific siRNA (Fig. 5).

## Discussion

MDSCs play an unquestionable role in tumor progression. Clinical studies in breast, colorectal, pancreatic, lung and gastric cancer patients demonstrated that increased MDSC level may be an independent prognostic factor for survival and may negatively correlate with responsiveness to chemotherapy [28]. However, our current understanding of MDSC biology is mainly based on experiments conducted using laboratory animals, and to date, only a minority of published studies has involved patients with spontaneous tumors [29], [30]. Considering the important role of MDSCs in tumor progression, more definitive clinical studies are required. Thus, the present paper includes both clinical data obtained from canine patients and data from *in vitro* investigations.

There is no current consensus regarding confirmed protein markers of MDSCs. In general, MDSCs express the common myeloid markers CD33 and CD11b [29], [30], but their immune suppressive functions can be identified by Gr1 expression [31]. Thus, to examine blood MDSC number by FACS, we used three markers: Gr1, CD33 and CD11b. Our results showed that the number of circulating MDSCs was significantly higher in stage I and II dogs with mammary cancer compared with healthy dogs; however, the number was the highest in stage III and IV dogs. This showed that the percentage of circulating MDSCs was associated with increased clinical stage and metastatic tumor burden. Although a similar trend was observed in human patients [28], [29], the number of MDSCs was lower than in our study (about 5% stage IV mammary tumor-bearing dogs compared with 15% in our study). All these results differed from data obtained in laboratory mice [32] that showed that circulating MDSCs constituted 50% of all the white blood cell population in stage IV animals. These differences can be related to the greater overall tumor burden reached in mice than in dogs or humans [30]. Moreover, in spontaneous tumors (such as in dogs or humans), the relationship between cancer and circulating MDSCs may be more complex than in mouse models. Nevertheless, in all these studies, the number of MDSCs increased linearly with tumor development. Therefore, the present study provides evidence that circulating MDSCs are clinically important in spontaneous tumor development and show similarities between dogs and humans. These findings may be very useful for further clinical trials of MDSC-targeting drugs that may involve canine model according to the “Comparative Oncology” program [17].

For many years, a role for MDSCs in cancer development has been linked with their suppressive function on the immune system [28]. However, Kujawski et al. (2008) showed that MDSCs could contribute to tumor angiogenesis. The authors revealed that MDSCs have highly activated STAT3, which upregulates the expression of various angiogenic genes [9]. Because many angiogenic proteins (e.g. VEGF-C) may simultaneously show immunosuppressive effects, MDSCs may interlink both functions [9]. Moreover, MDSCs (that have activated STAT3) may also increase STAT3 activity in cancer cells. Our previous report showed that the number of MDSCs in tumor tissues significantly correlated with p-STAT3 expression in cancer cells [31], [33]. However, the molecules mediating these interactions between both cell types have not yet been identified.

To explore the interactions between these cell types, we examined changes in gene expression in canine mammary tumor cells due to co-culture with MDSCs. To the best of our knowledge, our is the first microarray assay of these interactions. Analysis of the regulated gene list showed a significant increase in the expression of *Il-28ra*. Because IL-28 (IFN-λ) activates STAT3 [27], we decided to explore whether it functions in signaling between MDSCs and tumor cells. We showed that MDSCs secreted IL-28, and IL-28 expression in stage III and IV mammary tumor-bearing dogs was significantly higher than in healthy dogs or stage I or II canine patients. The functional significance underlying its dramatically increased expression in MDSCs of patients with advanced tumors is an interesting question that requires further investigation. We confirmed increased expression of IL-28RA at mRNA and protein levels in tumor cells co-cultured with MDSCs or treated with IL-28. These effects caused alterations in downstream signaling, including increased STAT3 phosphorylation, VEGV-C and SEMA3B expression, with the opposite effect in cells with IL-28RA knockdown. These molecular changes were associated with alterations in biological processes. Our results showed that tumor cells treated with IL-28 (before the experiment or before and during the experiment) for 6 h induced 3D vessel formation by endothelial cells. This may be related with an IL-28-mediated increase in the expression of VEGF-C and IL-18 [35] in tumor cells. Interestingly, co-culture of HUVECs with control cancer cells or with IL-28 alone for 6 h induced slight endothelial cell branching. It is very probable that longer stimulation would induce angiogenesis as well however it also shows that IL-28 stimulates cancer cells to secretion of angiogenic factors. Thus, MDSCs secrete IL-28 and then induce tumor angiogenesis mainly through an increase in secretion of angiogenic proteins in cancer cells. Co-culture of HUVECs with tumor cells with knockdown of IL-28RA completely abolished their angiogenic properties. These results suggest that the interactions among tumor cells, MDSCs, and endothelial cells are complex but essential for angiogenesis. However, each cell type may contribute differently at various stages of tumor development. These results suggest that IL-28 may be an interesting target for further investigation and therapies.

Because STAT3 influence cancer cell ability for invasion and migration [34] and microarray analysis in canine mammary tumor cells showed downregulation of *Keratin 17* due to MDSC co-culture, we examined a role of IL-28 in EMT [36]. Our investigation confirmed increased vimentin expression and decreased cytokeratin expression in neoplastic cells due to IL-28 treatment. Knockdown of IL-28RA caused slight increase in cytokeratin and decrease in vimentin expression. Moreover, we showed that treatment of cancer cells with IL-28 changed their shape (to spindle-like, in contrary to control cells and siRNA-treated cells forming colonies) and growth characteristics on Matrigel matrix, increasing their invasiveness what additionally confirms IL-28-induced EMT. All that data together indicated that MDSCs induced EMT in tumor cells, a crucial step to cancer invasiveness and metastasis [37]. The further assays conducted in Boyden chambers confirmed an important role of IL-28/IL-28RA signaling for tumor cell invasion and migration. We showed that IL-28RA knockdown significantly decreased neoplastic cell invasion and migration; however treatment with IL-28 increased tumor ability to migration. To the best of our knowledge, ours is the first report showing an involvement of MDSCs in neoplastic cell EMT, invasion and migration. Previously, only one conference report suggested that MDSCs can stimulate fibroblasts to increase carcinoma invasion and metastasis [38].

Together, our study showed that the canine model may be a good alternative for clinical trials of drugs targeting MDSCs or their interactions with cancer cells. Moreover, we showed for the first time that MDSCs communicate with tumor cells via IL-28/IL-28RA (IFN-λ) signaling activating STAT3 and thus stimulating angiogenesis, cancer cell EMT, invasion and migration. These conclusions are based on clinical and *in vitro* data, and thus further studies are required to examine the role of IL-28/IL-28RA signaling in the tumor microenvironment of different experimental models.

## Supporting Information

Figure S1 **Relative changes in gene and protein expression in canine mammary tumor cells due to their co-culture with MDSCs,** ***il-28ra*** **siRNA either IL-28 treatment.** A. Fold changes (based on SybrGreen fluorescence) of examined genes in CMT-U27, CMT-U309, and P114 canine mammary neoplastic cells cultured in control conditions or co-cultured with MDSCs. Analysis of variance and Tukey's test were applied (GraphPad Prism 5.0, USA); the values differed significantly (p<0.05) were marked as *, whereas values differed highly significant (p<0.01 or p<0.001) were marked as ** or ***, respectively. B. The level of examined proteins (by Western blot) in CMT-U27, CMT-U309 and P114 control cells or cells treated *il-28ra* siRNA either IL-28 was expressed as IOD (**I**ntegrated **O**ptical **D**ensity) in arbitrary units with the value obtained using the Odyssey Infrared Imaging System (LI-COR Inc., USA). The results are expressed as the mean ±SD. The **ANOVA +** Tukey post-hoc test were applied (Graph Pad v. 5.0), the values differed significantly (p**<**0.05) were marked as *, whereas values differed highly significant (p**<**0.01 or p<0.001) were marked as ** or ***, respectively.
(TIF)

Table S1 **The list of genes (and their fold change, FC) regulated in canine mammary tumor cell lines CMT-U27, CMT-U309 and P114 due to their co-culture with MDSCs.** The Statistical analyses were performed using Future Extraction, Gene Spring software (Agilent) and BRB ArrayTools (http://linus.nci.nih.gov/BRB-ArrayTools.html, Biometric Research Branch, US National Cancer Institute). Intensities were normalized using average factors scaled to the median array intensities over the entire array by using the median array as a reference. Probe sets that yielded a maximal normalized nonlog intensity value of 10 or less were filtered out from further analysis. Class comparsion analysis using two-sided Student t-tests identified mRNAs that were differentially expressed between signal and control samples (p<0.05; FC>2.0).
(DOC)
