# Supplementary figures and images for: MDSCs Mediate Angiogenesis and Predispose Canine Mammary Tumor Cells for Metastasis via IL-28/IL-28RA (IFN-λ) Signaling
Source: PLoS One. 2014 Jul 30;9(7):e103249. doi: 10.1371/journal.pone.0103249 (PMC4116234; doi:10.1371/journal.pone.0103249)

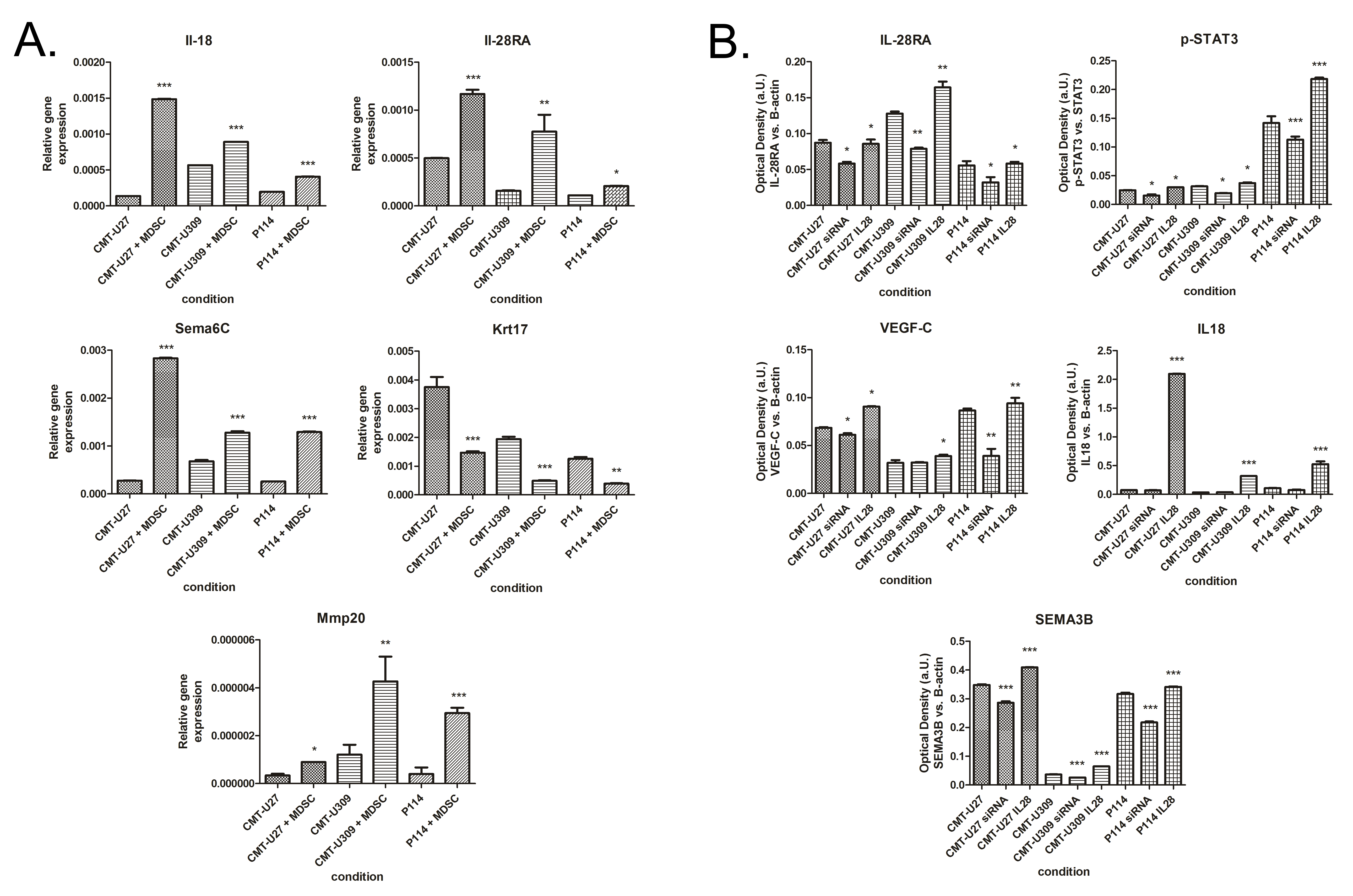

Supplement: Figure S1 — Relative changes in gene and protein expression in canine mammary tumor cells due to their co-culture with MDSCs, il-28ra siRNA either IL-28 treatment. A. Fold changes (based on SybrGreen fluorescence) of examined genes in CMT-U27, CMT-U309, and P114 canine mammary neoplastic cells cultured in control conditions or co-cultured with MDSCs. Analysis of variance and Tukey's test were applied (GraphPad Prism 5.0, USA); the values differed significantly (p<0.05) were marked as *, whereas values differed highly significant (p<0.01 or p<0.001) were marked as ** or ***, respectively. B. The level of examined proteins (by Western blot) in CMT-U27, CMT-U309 and P114 control cells or cells treated il-28ra siRNA either IL-28 was expressed as IOD (Integrated Optical Density) in arbitrary units with the value obtained using the Odyssey Infrared Imaging System (LI-COR Inc., USA). The results are expressed as the mean ±SD. The ANOVA + Tukey post-hoc test were applied (Graph Pad v. 5.0), the values differed significantly (p<0.05) were marked as *, whereas values differed highly significant (p<0.01 or p<0.001) were marked as ** or ***, respectively. (TIF) [file pone.0103249.s001.tif]
