# Supplementary material for: MDSCs Mediate Angiogenesis and Predispose Canine Mammary Tumor Cells for Metastasis via IL-28/IL-28RA (IFN-λ) Signaling
Source: PLoS One. 2014 Jul 30;9(7):e103249. doi: 10.1371/journal.pone.0103249 (PMC4116234; doi:10.1371/journal.pone.0103249)
Supplement: Table S1 — The list of genes (and their fold change, FC) regulated in canine mammary tumor cell lines CMT-U27, CMT-U309 and P114 due to their co-culture with MDSCs. The Statistical analyses were performed using Future Extraction, Gene Spring software (Agilent) and BRB ArrayTools (http://linus.nci.nih.gov/BRB-ArrayTools.html, Biometric Research Branch, US National Cancer Institute). Intensities were normalized using average factors scaled to the median array intensities over the entire array by using the median array as a reference. Probe sets that yielded a maximal normalized nonlog intensity value of 10 or less were filtered out from further analysis. Class comparsion analysis using two-sided Student t-tests identified mRNAs that were differentially expressed between signal and control samples (p<0.05; FC>2.0). (DOC) [file pone.0103249.s002.doc]

**Table S**1.

| No. | Regulation | FC | GeneSymbol | Description | |
| --- | --- | --- | --- | --- | --- |
| 1 | up | 3.074035 | ATP8B4 | ATPase, class I, type 8B, member 4 | |
| 2 | up | 2.906459 | GTF2A1L | Canis lupus familiaris general transcription factor IIA, 1-like (GTF2A1L) | |
| 3 | up | 2.864406 | ABCC12 | ATP-binding cassette, sub-family C (CFTR/MRP), member 12 | |
| 4 | up | 2.788163 | ACSM5 | acyl-CoA synthetase medium-chain family member 5 | |
| 5 | up | 2.787596 | MURC | muscle-related coiled-coil protein | |
| 6 | up | 2.765492 | APOB | apolipoprotein B | |
| 7 | up | 2.739976 | CPNE4 | copine IV | |
| 8 | up | 2.737685 | TMPRSS11F | transmembrane protease, serine 11F | |
| 9 | up | 2.682881 | ALDOB | Fructose-bisphosphate aldolase B (Liver-type aldolase), transcript variant 4 | |
| 10 | up | 2.655458 | TMC1 | transmembrane channel-like 1 | |
| 11 | up | 2.65377 | SLC22A25 | UST1-like organic anion transporter, transcript variant 1 | |
| 12 | up | 2.648698 | DMD | dystrophin (muscular dystrophy, Duchenne and Becker types) | |
| 13 | up | 2.593141 | FETUB | fetuin B | |
| 14 | up | 2.524806 | FGB | Fibrinogen beta chain Fragment | |
| 15 | up | 2.506275 | CCDC30 | coiled-coil domain containing 30 | |
| 16 | up | 2.495513 | IL-29 | Interleukin-29 precursor (IL-29) (Interferon lambda-1) (IFN-lambda-1) | |
| 17 | up | 2.476382 | TNNI3K | TNNI3 interacting kinase (TNNI3K) | |
| 18 | up | 2.450198 | CCDC83 | coiled-coil domain containing 83 | |
| 19 | up | 2.448924 | MMP20 | matrix metallopeptidase 20 | |
| 20 | up | 2.447642 | SIRPB1 | signal-regulatory protein beta 1 |  |
| 21 | up | 2.446149 | PLN | Canis lupus familiaris phospholamban (PLN) | |
| 22 | up | 2.445868 | TMCO3 | transmembrane and coiled-coil domains 3 | |
| 23 | up | 2.43231 | IL28RA | interleukin 28 receptor, alpha (interferon, lambda receptor) | |
| 24 | up | 2.400443 | cOR51H5 | 51H5 olfactory receptor protein Fragment | |
| 25 | up | 2.395421 | UCMA | upper zone of growth plate and cartilage matrix associated | |
| 26 | up | 2.38437 | TRDN | Canis lupus familiaris triadin (TRDN) | |
| 27 | up | 2.372447 | CAPN13 | calpain 13 | |
| 28 | up | 2.369695 | ZYX | zyxin | |
| 29 | up | 2.363653 | IL18 | Canis lupus familiaris interleukin 18 (interferon-gamma-inducing factor) (IL18) | |
| 30 | up | 2.363137 | KLB | klotho beta | |
| 31 | up | 2.361726 | MYH2 | Canis lupus familiaris myosin, heavy chain 2, skeletal muscle, adult (MYH2), | |
| 32 | up | 2.345666 | GPR151 | G protein-coupled receptor 151 | |
| 33 | up | 2.341518 | DDX43 | DEAD (Asp-Glu-Ala-Asp) box polypeptide 43 | |
| 34 | up | 2.333239 | CAFA-T2R7 | Canis lupus familiaris bitter taste receptor Cafa-T2R7 (CAFA-T2R7) | |
| 35 | up | 2.321282 | LMO3 | LIM-only protein 3 (Neuronal specific transcription factor DAT1), transcript variant 7 | |
| 36 | up | 2.319483 | GPR18 | G protein-coupled receptor 18 | |
| 37 | up | 2.309899 | SIRPB2 | signal-regulatory protein beta 2 | |
| 38 | up | 2.30124 | PYGB | Glycogen phosphorylase, brain form, transcript variant 1 | |
| 39 | up | 2.290391 | FGF14 | fibroblast growth factor 14 | |
| 40 | up | 2.28945 | SPARCL1 | SPARC-like 1 (hevin) | |
| 41 | up | 2.28184 | ACTN2 | actinin, alpha 2, transcript variant 7 | |
| 42 | up | 2.274676 | CFL1 | Cofilin-1 (Cofilin, non-muscle isoform) (18 kDa phosphoprotein) (p18), transcript variant 2 | |
| 43 | up | 2.27383 | MLF2 | myeloid leukemia factor 2 | |
| 44 | up | 2.273008 | ARHGEF10L | Rho guanine nucleotide exchange factor (GEF) 10-like | |
| 45 | up | 2.262348 | NLRP10 | NLR family, pyrin domain containing 10 | |
| 46 | up | 2.255762 | SOX6 | SRY (sex determining region Y)-box 6 | |
| 47 | up | 2.252456 | YPEL4 | yippee-like 4 (Drosophila) | |
| 48 | up | 2.240016 | SCUBE2 | signal peptide, CUB domain, EGF-like 2 | |
| 49 | up | 2.230896 | SPACA1 | sperm acrosome associated 1 | |
| 50 | up | 2.230369 | FSIP1 | fibrous sheath interacting protein 1 | |
| 51 | up | 2.227014 | PCYOX1L | prenylcysteine oxidase 1 like | |
| 52 | up | 2.221959 | NEFM | neurofilament, medium polypeptide | |
| 53 | up | 2.214348 | ADCY2 | adenylate cyclase 2 (brain) | |
| 54 | up | 2.207355 | CBLN1 | cerebellin 1 precursor | |
| 55 | up | 2.197241 | DRGX | dorsal root ganglia homeobox | |
| 56 | up | 2.18702 | PLXNA4 | plexin A4 | |
| 57 | up | 2.181463 | CLRN3 | clarin 3 | |
| 58 | up | 2.178591 | KCNMB2 | potassium large conductance calcium-activated channel, subfamily M, beta member 2 | |
| 59 | up | 2.173789 | SEMA6C | sema domain, transmembrane domain (TM), and cytoplasmic domain, (semaphorin) 6C | |
| 60 | up | 2.17132 | CDH20 | Cadherin 20 | |
| 61 | up | 2.16977 | ARX | aristaless related homeobox (ARX) | |
| 62 | up | 2.167824 | EML5 | echinoderm microtubule associated protein like 5 | |
| 63 | up | 2.167218 | FMO3 | Canis lupus familiaris flavin containing monooxygenase 3 (FMO3) | |
| 64 | up | 2.162804 | BCO2 | beta-carotene oxygenase 2 | |
| 65 | up | 2.158243 | DDX39B | Canis lupus familiaris DEAD (Asp-Glu-Ala-Asp) box polypeptide 39B (DDX39B) | |
| 66 | up | 2.152697 | ZSWIM5 | zinc finger, SWIM-type containing 5 | |
| 67 | up | 2.152433 | GAP43 | growth associated protein 43 | |
| 68 | up | 2.148493 | MAP6 | microtubule-associated protein 6 | |
| 69 | up | 2.146782 | Mar-01 | membrane-associated ring finger (C3HC4) 1 | |
| 70 | up | 2.139508 | COL24A1 | collagen, type XXIV, alpha 1 | |
| 71 | up | 2.13871 | PRKAR1B | protein kinase, cAMP-dependent, regulatory, type I, beta | |
| 72 | up | 2.137981 | SEC14L3 | SEC14-like 3 (S. cerevisiae) | |
| 73 | up | 2.128108 | ARHGAP24 | Rho GTPase activating protein 24 | |
| 74 | up | 2.128045 | SLC29A1 | Canis lupus familiaris solute carrier family 29 (nucleoside transporters), member 1 (SLC29A1), nuclear gene encoding mitochondrial protein | |
| 75 | up | 2.127272 | MAEL | maelstrom homolog (Drosophila) | |
| 76 | up | 2.124895 | CCL26 | Canis lupus familiaris chemokine (C-C motif) ligand 26 (CCL26) | |
| 77 | up | 2.122682 | SIAE | cytosolic sialic acid 9-O-acetylesterase homolog, transcript variant 1 | |
| 78 | up | 2.118898 | MGAT4C | mannosyl (alpha-1,3-)-glycoprotein beta-1,4-N-acetylglucosaminyltransferase, isozyme C (putative) | |
| 79 | up | 2.118572 | RBP3 | Interphotoreceptor retinoid-binding protein Fragment | |
| 80 | up | 2.102341 | PTH | Canis lupus familiaris parathyroid hormone (PTH) | |
| 81 | up | 2.096335 | ARPP21 | cAMP-regulated phosphoprotein, 21kDa | |
| 82 | up | 2.089426 | PVALB | parvalbumin | |
| 83 | up | 2.089405 | MAGT1 | magnesium transporter 1 | |
| 84 | up | 2.086929 | IL2 | Canis lupus familiaris interleukin 2 (IL2) | |
| 85 | up | 2.08638 | MYPN | myopalladin | |
| 86 | up | 2.085347 | PDCL2 | phosducin-like 2 | |
| 87 | up | 2.084499 | GIMAP4 | GTPase, IMAP family member 4 | |
| 88 | up | 2.078318 | SLC25A39 | solute carrier family 25, member 39 | |
| 89 | up | 2.076062 | LAPTM5 | PREDICTED: Canis familiaris similar to Lysosomal-associated multitransmembrane protein (Retinoic acid-inducible E3 protein) (HA1520) | |
| 90 | up | 2.073849 | SOX30 | SRY (sex determining region Y)-box 30 | |
| 91 | up | 2.071693 | PCDHAC2 | Protocadherin alpha C2 precursor (PCDH-alpha-C2), transcript variant 1 | |
| 92 | up | 2.061004 | ASB2 | Canis familiaris similar to ankyrin repeat and SOCS box-containing protein 2 | |
| 93 | up | 2.059515 | TMPRSS11D | transmembrane protease, serine 11D | |
| 94 | up | 2.054925 | MSH4 | mutS homolog 4 (E. coli) | |
| 95 | up | 2.043356 | FGF12 | fibroblast growth factor 12 | |
| 96 | up | 2.033491 | GIMAP8 | GTPase, IMAP family member 8 | |
| 97 | up | 2.030081 | TXLNB | taxilin beta | |
| 98 | up | 2.029255 | TDRD6 | tudor domain containing 6 | |
| 99 | up | 2.027206 | TRPM1 | transient receptor potential cation channel, subfamily M, member 1 | |
| 100 | up | 2.021542 | MYH1 | Canis lupus familiaris myosin, heavy chain 1, skeletal muscle, adult (MYH1) | |
| 101 | up | 2.016797 | IL7 | Canis lupus familiaris interleukin 7 (IL7) | |
| 102 | up | 2.015542 | STEAP4 | STEAP family member 4 | |
| 103 | up | 2.010653 | NOSTRIN | nitric oxide synthase trafficker | |
| 104 | up | 2.010567 | CLUL1 | Canis lupus familiaris clusterin-like 1 (retinal) (CLUL1) | |
| 105 | up | 2.008676 | STMN4 | stathmin-like 4 | |
| 106 | up | 2.004662 | RNF19B | ring finger protein 19B | |
| 107 | up | 2.002299 | DEFB3L | Canis lupus familiaris beta-defensin-like peptide 3 (DEFB3L), mRNA | |
| 108 | down | -2.00509 | GPR97 | G protein-coupled receptor 97 | |
| 109 | down | -2.01082 | SCN2A | sodium channel, voltage-gated, type II, alpha subunit | |
| 110 | down | -2.01719 | DDX25 | Canis familiaris similar to DEAD (Asp-Glu-Ala-Asp) box polypeptide 25 | |
| 111 | down | -2.02192 | RBMS3 | Canis familiaris similar to RNA binding motif, single stranded interacting protein 3 isoform 3, transcript variant 6 | |
| 112 | down | -2.03246 | IL17RE | interleukin 17 receptor E | |
| 113 | down | -2.0346 | CfOLF4 | Canis lupus familiaris olfactory receptor 4 (CfOLF4) | |
| 114 | down | -2.03542 | GPR37 | G protein-coupled receptor 37 (endothelin receptor type B-like) | |
| 115 | down | -2.0555 | SOCS3 | Canis lupus familiaris suppressor of cytokine signaling 3 (SOCS3) | |
| 116 | down | -2.0604 | KRT17 | Keratin 17 Fragment | |
| 117 | down | -2.06718 | LEAP2 | liver expressed antimicrobial peptide 2 | |
| 118 | down | -2.06972 | ENPP2 | ectonucleotide pyrophosphatase/phosphodiesterase 2 | |
| 119 | down | -2.07351 | OLFML2A | PREDICTED: Canis familiaris similar to olfactomedin-like 2A | |
| 120 | down | -2.07643 | OTOS | otospiralin | |
| 121 | down | -2.10861 | SNAI3 | snail homolog 3 (Drosophila) | |
| 122 | down | -2.13048 | RAB3A | RAB3A, member RAS oncogene family | |
| 123 | down | -2.13185 | PAQR8 | progestin and adipoQ receptor family member VIII | |
| 124 | down | -2.13549 | TNNC1 | iv33d11.g1 Left Cardiac Ventricle (DOGEST7) | |
| 125 | down | -2.1422 | CLN8 | Canis lupus familiaris ceroid-lipofuscinosis, neuronal 8 (epilepsy, progressive with mental retardation) (CLN8) | |
| 126 | down | -2.1544 | CAP2 | PREDICTED: Canis familiaris similar to adenylyl cyclase-associated protein 2, transcript variant 3 | |
| 127 | down | -2.16491 | TMEM42 | transmembrane protein 42 | |
| 128 | down | -2.20595 | TFF3 | Trefoil factor 3 Precursor (Intestinal trefoil factor) | |
| 129 | down | -2.24468 | RASGRP4 | RAS guanyl releasing protein 4 | |
| 130 | down | -2.25918 | SLC27A1 | solute carrier family 27 (fatty acid transporter), member 1 | |
| 131 | down | -2.28331 | ABCC8 | Sulfonylurea receptor 1 Fragment | |
| 132 | down | -2.29223 | GLDN | collomin | |
| 133 | down | -2.34516 | TNC | Canis lupus familiaris tenascin C (TNC) | |
| 134 | down | -2.35878 | SLC46A2 | Canis lupus familiaris solute carrier family 46, member 2 (SLC46A2) | |
| 135 | down | -2.3679 | PVRL1 | Prr1 protein Fragment | |
| 136 | down | -2.37113 | PGB | progastricsin (pepsinogen C) (PGB) | |
| 137 | down | -2.7005 | LCTL | lactase-like | |
